# *Pantoea eucrina* : Catheter‐Related Bloodstream Infection in a Woman With Short Bowel Syndrome

**DOI:** 10.1002/ccr3.70103

**Published:** 2025-01-10

**Authors:** Ida Saksenborg Kølle, Lars Vinter‐Jensen, Morten Eneberg Nielsen, Hans Linde Nielsen

**Affiliations:** ^1^ Department of Gastroenterology and Hepatology Aalborg University Hospital Aalborg Denmark; ^2^ Department of Clinical Medicine Aalborg University Aalborg Denmark; ^3^ Department of Clinical Microbiology Aalborg University Hospital Aalborg Denmark; ^4^ Department of Chemistry and Bioscience Aalborg University Aalborg Denmark

**Keywords:** bacteremia, bloodstream infection, Enterobacterales, Pantoea species, sepsis, short bowel syndrome

## Abstract

*Pantoea eucrina* , a member of the Erwiniaceae family, is a rarely reported human pathogen primarily associated with plants. This study presents a documented case of catheter‐related bloodstream infection caused by *P. eucrina* in a 60‐year‐old female receiving home parenteral nutrition. Despite presenting with only minor clinical symptoms, blood cultures from both central and peripheral sites confirmed the presence of *P. eucrina* , identified by matrix‐assisted laser desorption/ionization time‐of‐flight mass spectrometry (MALDI‐TOF MS) and whole‐genome sequencing. The infection was successfully cleared following a 13‐day course of gentamicin lock therapy and intravenous piperacillin–tazobactam, after which the catheter was removed.

## Summary

-
*Pantoea eucrina* infections are rare.
- We report a catheter‐related bloodstream infection in a 60‐year‐old woman on home parenteral nutrition.
- Positive blood cultures grew *P. eucrina* .
- Gentamicin lock therapy and intravenous piperacillin–tazobactam, followed by catheter removal ensured successful management.

## 1 Introduction

The genus *Pantoea* encompasses a diverse group of gram‐negative bacteria characterized by yellow pigmentation and rod‐shaped morphology, belonging to the *Erwiniaceae* family [1]. Currently, the genus *Pantoea* comprises 20 validated species [2], with *Pantoea agglomerans* (formerly *Enterobacter agglomerans* ) identified as the type species and recognized as the primary causative agent in the majority of human infections, emerging as an opportunistic pathogen [3, 4].

Early research on *Pantoea* primarily focused on its parasitic association with plant hosts, where it facilitated plant diseases through pathogenicity factors such as the type III secretion system, quorum‐sensing systems, and exopolysaccharides [4]. In human infections, *Pantoea* species have been isolated from both immunocompetent and immunocompromised patients, often associated with various clinical manifestations, including wound, blood, and urinary tract infections, and contaminated catheters [5].

*Pantoea eucrina* , initially described by Brady et al., was first identified in humans but subsequently isolated from diverse sources, including a trash can surface and the rhizospheric soil of wheat, exhibiting characteristics of a plant‐growth‐promoting bacterium [5, 6]. In the human context, this exceedingly rare pathogen has been isolated from diverse clinical sources, including blood, spinal fluid, cysts, urine, and trachea, with documented cases reported from the United States, France, and Italy [5, 6, 7, 8].

Catheter‐related bloodstream infections (CRBSIs) pose a life‐threatening complication for individuals undergoing home parenteral nutrition (HPN). Overall, the CRBSI rate has ranged between 0.38 and 4.58 episodes/1000 catheter days (median 1.31), and gram‐positive bacteria of the human skin microbiota caused more than half of the infections [9]. Here, we describe the first Danish case of *P. eucrina* CRBSI in a patient requiring HPN.

## 2 Case Presentation

A 60‐year‐old woman receiving HPN underwent evaluation in an outpatient setting in May 2023. Blood tests revealed elevated levels of C‐reactive protein (CRP) at 95 mg/L, but a normal white blood cell count (WBC) of 6.0 × 10^9^/L. Despite being asymptomatic, further investigation was initiated for suspected CRBSI caused by unspecified inflammation. Blood cultures were obtained from both the central venous catheter and a peripheral venipuncture. Following assessment, she was discharged with instructions to monitor for signs of infection. The following day, she was admitted to the Department of Gastroenterology at Aalborg University Hospital, Denmark, because of positive blood cultures showing motile gram‐negative rods, later identified as *P. eucrina* . She was afebrile, with a pulse of 100 beats per minute, and no other abnormal objective findings were noted.

In 2009, the patient was diagnosed with uterine cancer and underwent a hysterectomy with lymph node removal. She later required a right hemicolectomy in 2013 because of metastatic spread to the small intestine, necessitating chemotherapy and radiotherapy. She also suffered from chronic noninfectious diarrhea and experienced significant weight loss, dropping from 96 to 30 kg after her last surgery. In February 2023, she began HPN and fluid support via a Hickman catheter. Despite her complex medical condition, she weighed 46 kg during outpatient follow‐up in May 2023. For a detailed overview of her medical history, see Table 1.

**TABLE 1:** Key diagnoses, treatments, and complications in the patient's medical history.

Month and year	Diagnosis and treatment
December 2009	Diagnosed with uterine cancer (squamous cell carcinoma) and underwent a radical hysterectomy with lymph node removal from the groin
March 2013	Metastases to the intestine necessitated a right hemicolectomy, followed by adjuvant chemotherapy and radiotherapy (45 Gy/25 fractions targeting the pelvis and para‐aortic lymph nodes)
May 2014	Nonspecific chronic inflammation of the colon and chronic diarrhea, likely resulting from radiation damage caused by her treatment
January 2018	Laparoscopic sigmoidostomy performed because of radiation sequelae, complicated by intestinal obstruction and fistula formation
February 2018	Stoma revision performed because of poor function
July 2020	Diagnosed with insulin‐dependent diabetes mellitus and chronic kidney disease
December 2021	Diagnosed with chronic pancreatitis
May 2022	Small bowel obstruction treated surgically with adhesiolysis and mobilization of the twisted intestine
June 2022	Diagnosed with exocrine pancreatic insufficiency
February 2023	Third‐degree atrioventricular block caused by electrolyte imbalance, treated with pacemaker placement
January 2023	Bowel insufficiency requiring initiation of parenteral nutrition via a Hickman catheter
May 2023	Bacteremia caused by *Pantoea eucrina*

## 3 Treatment and Outcome

The Hickman catheter was instilled with 5 mL of gentamicin solution (1 mg/mL), corresponding to a daily dose of 5 mg, and intravenous therapy with piperacillin–tazobactam was initiated at a dosage of 4 g + 0.5 g, three times daily. Within the first 24 h, this regimen was transitioned to a continuous daily infusion of 12 g + 1.5 g piperacillin–tazobactam administered via a CADD pump. Both treatments were sustained for a total duration of 13 days.

Subsequent to treatment initiation, normalization of CRP levels was observed. A follow‐up blood culture obtained on Day 6 yielded negative results. Consequently, a decision was made to extract the Hickman catheter, discontinue fluid and nutritional support, and evaluate her capacity to maintain weight gain independently. Throughout the posttreatment follow‐up period, she did not require HPN and maintained a stable weight of 46 kg. Upon discharge, she underwent regular monthly blood tests, encompassing biochemical markers to assess fluid balance, renal function, and indicators of infection, in conjunction with consultations with a dietitian. She continued to maintain her weight without the need for HPN, remaining at approximately 46 kg as of February 2024.

## 4 Methods

Two standard blood cultures (using two BD BACTEC Plus Aerobic medium and one BD BACTEC Lytic Anaerobic medium glass culture vials) were obtained from both the Hickman catheter and a peripheral venipuncture upon submission. Subsequently, these culture vials were incubated in the BACTEC FX Top instrument (Becton Dickinson AB, Stockholm, Sweden). After 1 day of incubation, we observed the growth of motile, gram‐negative rods in all three blood culture bottles obtained from the Hickman catheter. The time to detection (TTD) for these cultures was 4.6 h and 5.2 h for the two aerobic bottles, respectively, and 7.9 h for the anaerobic bottle. In contrast, the bottles obtained from the peripheral site exhibited a longer TTD: 11.4 h and 11.9 h for the two aerobic bottles, and 35.1 h for the anaerobic bottle.

Sub‐cultivation was performed on solid agar medias and colonies were identified as *P. eucrina* by use of the matrix‐assisted laser desorption ionization–time of flight (MALDI Biotyper 3.1, Bruker Daltonics Microflex LT, MBT 6903 MSP Library) with a score of 2.26.

Antibiotic susceptibility tests (AST) of *P. eucrina* were conducted according to EUCAST breakpoints for Enterobacterales (version 13.1). Minimum inhibitory concentrations (MICs) were determined using Etest (BioMérieux, Marcy l'Etoile, France). The isolate was reported as susceptible to all antibiotics tested (using standard dosing regimens), including ampicillin (MIC: 2 mg/L), cefotaxime (MIC: 0.064 mg/L), meropenem (MIC: 0.032 mg/L), gentamicin (MIC: 0.25 mg/L), and ciprofloxacin (MIC: 0.032 mg/L). For piperacillin–tazobactam, where no Etest was available, disk diffusion (disk content, 30‐6 μg) demonstrated an inhibition zone of 30 mm.

Next, we extracted bacterial DNA with the DNeasy 96 PowerSoil Pro QIAcube HT Kit (cat. no. 47021, Qiagen, Hilden, Germany) on a QIAcube HT system (cat. no. 9001896). DNA was prepared for sequencing using the Rapid Barcoding Kit (SQK‐RBK114.96) from Oxford Nanopore Technologies, with 50 ng per sample as input. The library was sequenced on a Promethion flowcell (FLO‐PRO114M) on the P24 platform. Basecalling was conducted with Dorado (v0.4.2) and reads with mean quality > Q10 and read length > 2 kb were processed using an in‐house pipeline for genome assembly and QC (github.com/MortenEneberg/Nanopore_WGS). In short, reads were assembled with Flye (v2.3.2, settings: ‐‐nano‐hq) [10], the assembly quality assessed with CheckM2 (v1.0.1), https://paperpile.com/c/MJMLSc/Q1mJ [11], and the assembly ID found with GTDB‐Tk classify_wf (v2.3.2, database release 214) (https://paperpile.com/c/MJMLSc/RGU9) [12]. Resistance genes were annotated using ABRicate (v1.0.1, https://github.com/tseemann/abricate) with default settings and the NCBI database https://paperpile.com/c/MJMLSc/2Cg1 [13].

Using whole‐genome sequencing (WGS) revealed that the isolate had a total genome size of 4.05 Mb and a GC content of 56%, and yielded a circular chromosome with six plasmids. The genome was identified as *P. eucrina* (98.25 ANI to *P. eucrina* reference: GCF_002095385.1), confirming the identification by MALDI‐TOF. Moreover, one of the plasmids was identified as the Large Pantoea Plasmid (LPP‐1) with a size of 369 kb, in accordance with the literature [14]. Only one resistance gene was identified in the genome as *oqxB9* (reference gene: NG_050458.1) located on the chromosome and encoding a multidrug efflux RND transporter permease subunit with unknown specificity. Importantly, no chromosomally encoded β‐lactamase genes were detected.

## 5 Discussion

This case report presents the first documented instance of *P. eucrina* CRBSI in Denmark and only the fifth documented case in the literature [7, 8]. The patient, a 60‐year‐old woman with chronic pancreatitis, chronic kidney failure, and insulin‐dependent diabetes, was undergoing HPN via a Hickman catheter. Despite minimal symptoms, she had elevated CRP levels and positive blood cultures from both central and peripheral sites, confirming a CRBSI caused by *P. eucrina* . The infection was successfully treated with gentamicin lock (5 mL of 1 mg/mL solution) and piperacillin–tazobactam (4 g + 0.5 g t.i.d. initially, then switched to continuous infusion of 12 g + 1.5 g/day via a CADD pump), followed by catheter removal. Despite her significant medical challenges, the patient maintained stable weight and functionality without further HPN.

The genus *Pantoea*, predominantly recognized as plant pathogens, has been increasingly identified as opportunistic human pathogens [5, 6, 7, 8]. *Pantoea* species are frequently isolated from environmental sources such as soil, fruits, and vegetables, and occasionally from human and animal microbiomes. Among these, *P. eucrina* is notably rare, with limited reports of its involvement in human infections, including bacteremia, cerebrospinal fluid infections, cyst infections, urinary tract infections, and tracheal infections [6, 7, 8].

HPN is essential for patients with intestinal failure but significantly increases the risk of CRBSI, a potentially life‐threatening complication [9]. According to ESPEN guidelines—ESPEN being the European Society for Clinical Nutrition and Metabolism, originally established in 1980 as the European Society for Parenteral and Enteral Nutrition—CRBSI is a clinical diagnosis identified by a positive catheter culture upon removal, paired blood cultures from a peripheral vein and the catheter, or clinical symptoms of sepsis [15, 16]. The pathogenesis of CRBSI usually involves the migration of skin microbiota through the percutaneous entry site [16], with common pathogens including gram‐positive bacteria, such as *Staphylococcus* species, followed by gram‐negative bacteria and fungi [9]. Symptoms can range from severe sepsis to vague indicators like elevated CRP or leukocyte counts, particularly with *Staphylococcus epidermidis* . In this case, *P. eucrina* presented with minimal symptoms, consistent with other CRBSI cases that often show nonspecific symptoms like elevated inflammatory markers rather than overt sepsis, highlighting the need for thorough investigation and timely diagnosis.

Casale et al. recently reported a 5‐year (2018–2023) single‐center study investigating 4,996 bloodstream infections with gram‐negative isolates [8]. *Pantoea* species accounted for 0.4% (*n* = 19) from 19 different patients, including five pediatric cases. *P. agglomerans* was the most frequently detected (45%; *n* = 9), followed by *P. septica* (30%; *n* = 6) and *P. eucrina* (15%; *n* = 3). Catheter‐related infections were identified in 4 of 14 adult cases (28.6%) and two of five pediatric cases (40%). Adults were predominantly treated with monotherapy (71.4%), whereas combination therapy (60%) was preferred for pediatric patients. The most frequently prescribed antibiotic regimens were piperacillin–tazobactam (20%) in adults and meropenem‐ (40%) and aminoglycoside‐containing (40%) antibiotics in pediatric patients [8].

Antibiotic susceptibility testing of *P. eucrina* revealed susceptibility to a broad spectrum of antibiotics, including ampicillin, piperacillin–tazobactam, cefotaxime, ertapenem, ciprofloxacin, and gentamicin. This aligns with the findings of Casale et al., which reported high susceptibility of *P. eucrina* to most antibiotics.

Lotte et al. reported a similar case of CRBSI with *P. eucrina* in a younger patient with colon adenocarcinoma and congenital hypogammaglobulinemia, presenting with fever and abdominal pain [7]. In both cases, initial treatment involved intravenous piperacillin–tazobactam at a dose of 4.5 g three times daily. However, while Lotte et al. later switched to cefoxitin, in our case, piperacillin–tazobactam was continued for 13 days. Given that no chromosomally or plasmid‐encoded β‐lactamase genes were detected in our strain of *P. eucrina* and it was reported susceptible to all β‐lactams tested, the antimicrobial therapy could potentially have been more specifically tailored. In hindsight, a switch to intravenous ampicillin, which has a narrower spectrum of activity and potentially fewer collateral effects, might have been more appropriate if the specific involvement of *P. eucrina* had been better understood earlier in the treatment course. In both instances, catheter removal and patient discharge for follow‐up were deemed necessary.

The use of advanced diagnostic techniques, such as MALDI‐TOF mass spectrometry and WGS, was pivotal in accurately identifying the *P. eucrina* strain in this case [17, 18]. The application of MALDI‐TOF has significantly improved the identification of uncommon bacterial species, thereby enhancing diagnostic accuracy and informing appropriate treatment strategies. However, as demonstrated in the case by Lotte et al., even advanced techniques like MALDI‐TOF may sometimes require supplementary molecular methods for precise identification.

## 6 Conclusion

This case underscores the importance of considering rare pathogens such as *P. eucrina* in the differential diagnosis of CRBSI, particularly in patients with complex medical histories and indwelling catheters. The integration of advanced diagnostic tools like MALDI‐TOF and WGS into routine clinical practice is crucial for the accurate identification of uncommon pathogens, which can inform targeted therapeutic interventions and improve patient outcomes. Further studies are warranted to elucidate the pathogenic potential and epidemiological trends of *Pantoea* species in human infections.

## Author Contributions

**Ida Saksenborg Kølle:** data curation, investigation, validation, visualization, writing – original draft. **Lars Vinter‐Jensen:** conceptualization, data curation, writing – review and editing. **Morten Eneberg Nielsen:** investigation, methodology, writing – review and editing. **Hans Linde Nielsen:** conceptualization, data curation, formal analysis, investigation, methodology, writing – original draft, writing – review and editing.

## Ethics Statement

Personal data have been respected.

## Consent

Written informed consent was obtained from the patient to publish this report in accordance with the journal's patient consent policy.

## Conflicts of Interest

The authors declare no conflicts of interest.

## Data Availability

The data that support the findings of this study are available from the corresponding author upon reasonable request.
